# Prevalence of Undernutrition and Risk of Undernutrition in Overweight and Obese Older People

**DOI:** 10.3389/fnut.2022.892675

**Published:** 2022-05-05

**Authors:** Claire Sulmont-Rossé, Virginie Van Wymelbeke-Delannoy, Isabelle Maître

**Affiliations:** ^1^Centre des Sciences du Goût et de l'Alimentation, INRAE, Université Bourgogne Franche-Comté, Institut Agro, CNRS, Dijon, France; ^2^CHU Dijon Bourgogne F Mitterrand, Unité du Pôle Personnes Âgées, Dijon, France; ^3^Ecole Supérieure d'Agricultures (ESA), USC 1422 GRAPPE, INRAE, SFR 4207 QUASAV, Angers, France

**Keywords:** aged, body weight, body mass index, malnutrition, dependence

## Abstract

Older people with excess body weight are not spared from undernutrition. They may face appetite decline which may lead to insufficient nutrient intake. They also have a higher risk of developing chronic diseases which may have a negative impact on protein-anabolic pathways. The present study aimed to determine the prevalence of undernutrition in overweight and obese older people from a secondary analysis on data collected through two French surveys among people aged 65 or over (*n* = 782; 31% men; 65–103 years old). Undernutrition was assessed using the MNA screening tool (Mini-Nutritional Assessment). Results showed that 2% of the respondents with a BMI over 25 were undernourished (MNA score below 17/30) and 23% were at risk of undernutrition (MNA score of 17–23.5). Specifically, 18% of overweight and 29% of obese respondents were at risk of undernutrition. Taking into account the most recent French census data, it can be estimated that in France, around 1,7 million people aged over 65 with a BMI over 25 are undernourished or at risk of undernutrition. Given the worldwide increase in the number of overweight/obese individuals in the last few decades, further research will be needed to develop strategies to tackle nutritional risk in overweight/obese older adults.

## Introduction

The issue of overweight and obesity in the older population is set to become a major public health issue in the near future given the increasing number of overweight and obese individuals worldwide. In the United States, 76% of men and 73% of women aged 60 years and over are classified as being overweight (Body Mass Index (BMI) ≥ 25 kg/m^2^). Among them, 37% of men and 42% of women are classified as having obesity (BMI ≥ 30 kg/m^2^) (1). In the EU-27, overweight affects 63% and obesity affects 20% of adults aged 65 and over (59 and 17% of adults aged 75 and over) (2).

Obesity increases the risk of developing a wide range of diseases such metabolic diseases, cardiovascular diseases and certain types of cancer which may affect quality of life and functional status in older people (3). However, some studies have suggested that obesity is associated with reduced mortality from cardiovascular diseases in older adults compared to younger adults (4, 5). For instance, in people with heart failure, those with a BMI between 30 and 35 had lower mortality than those who would normally be considered an ideal weight (6). Several biological mechanisms have been proposed to explain this “obesity paradox” during the course of aging, but this phenomenon remained controversial among scientists. Two recent reviews have highlighted the fact that most of the studies on the “obesity paradox” have focused on the BMI, whereas other factors such as lean body mass, fat distribution or cardiac fitness may be gaining importance in determining survival in obese older adults (7, 8). Mezian et al. (9) noted that although obesity may be protective against metabolic diseases, it accelerates muscle loss and causes sarcopenia.

Although undernutrition is less common than obesity, this disorder is one of the biggest threats to the health and autonomy of older adults (10, 11). Undernutrition is caused by insufficient nutrient intake and/or compromised assimilation of nutrients (12). In the older population, undernutrition is associated with adverse functional and clinical outcomes. It leads to altered body composition (loss of muscle mass) and diminished biological function (impairment of immune function). It increases the risk of falls and fractures, and the risk of infectious episodes and hospital readmission (13, 14). Without care, undernutrition induces or worsens a state of frailty and dependence, and affects quality of life and life expectancy (15–17).

Older people with overweight or obesity are not spared from undernutrition. Like all older individuals, they are likely to face changes which may disrupt eating behavior and lead to a decline in appetite and food intake (18). These changes can be physiological (hormonal dysregulation, oral impairment, sensory decline…), psychological (depression, dementia…) or sociological (retirement, loss of income, loneliness…) (19). In addition, obese individuals have a higher risk of developing chronic diseases leading to organ failure, and a higher risk of cancer and infection. These health conditions often result in acute complications and hospitalization. Chronic and acute diseases have a negative impact on nutritional status, both through a negative impact on skeletal muscle protein-anabolic pathways and due to reduced appetite and nutrient intake (20). Finally, Fleury et al. (21) observed an average daily protein intake per kg body weight of 1.0 g for older people with normal weight status and 0.7 for obese older people when considering the recommendation of 1.2 g protein / kg body weight / day in older people (22). In the obese older people, insufficient protein intake may lead to sarcopenic obesity, which is characterized by skeletal-muscle atrophy and increased adiposity (23). Some evidence indicates that the coexistence of sarcopenia and obesity is associated with higher levels of metabolic disorders, functional decline and an increased risk of mortality compared with to sarcopenia or obesity alone (24, 25).

To the best of our knowledge, there are very few studies reporting prevalence data for undernutrition and risk of undernutrition in overweight and obese older adults. In Brazil, Klee Oehlschlaeger et al. (26) reported that 23% of seniors with a BMI over 25 kg/m^2^ were identified as at risk or undernourished by the MNA. In this study, respondents were community-dwelling seniors aged 60 or older and attending recreational or physical activity groups (*n* = 210), and 83% of respondents had excess weight (overweight and obesity). In Turkey, in seniors aged 65 and older with a BMI over 25 kg/m^2^ (*n* = 1,205), Özkaya and Gürbüz (27) reported that undernutrition and risk of malnutriton were respectively 19% and 31%. This study used the MNA-Short Form (28). Neither of these studies provided separate data for overweight and obese participants.

Consequently, the primary outcome of the present study was to provide prevalence data for undernutrition and risk of undernutrition for overweight and obese older people. The secondary outcome was to investigate the relationship between weight status and nutritional status, taking into account possible cofounders. We hypothesized that being underweight will be associated with a higher risk of undernutrition and that being overweight or obese will be associated with a lower risk of undernutrition compared to normal-weight respondents. However, given the worldwide increase in the number of overweight/obese individuals in the last few decades, we hypothesized that the number of overweight or obese older people being at risk of undernutrition will be significant. Data were derived from a secondary analysis on data collected through two French surveys conducted in 2011 and 2016 among people aged 65 years or over. In these surveys, undernutrition was assessed using the MNA, in line with the recommendations from the French Health Authority (29).

## Method

### Participants

The present study is a secondary analysis of data collected through two surveys conducted among French individuals aged 65 years or over: the AUPALESENS project in 2011 and the RENESSENS project in 2015. The primary outcome of the AUPALESENS survey was to compare the undernutrition risk between older people who delegated all or part of their meal-related activities (food purchasing, cooking) and older people who were still preparing their own meals. This survey included 559 people living at home without or with help and people living in nursing home. The primary outcome of the RENESSENS survey was to compare the undernutrition risk when meals were delegated to different third parties. Consequently, this second survey included 319 people with food help provided by a caregiver, a meals-on-wheels service or a nursing home. In both surveys, recruitment was achieved through advertisements in local newspapers, flyers in local senior associations and with support from local care facilities.

Sample characteristics and primary analyses are published in Van Wymelbeke-Delannoy et al. (30). For the present secondary analysis, we were able to combine the data from these two surveys seeing that the nutritional status, the weight status and the background variables were measured using the same methods. In this secondary analysis, four living arrangements were considered: (1) people living independently at home; (2) people living at home with help provided by a care-giver; (2) people living at home with a regular meals-on-wheels service; (4) people living in a nursing home. Inclusion criteria were the following: no acute illness at the time of the survey no food allergies; no prescribed diet and MMSE score of at least 21 to ensure reliable responses in the survey **(**Mini Mental State Examination; 31).

### Procedure

Each participant underwent two 60 to 90-min face-to-face interviews to collect nutritional and background data. Nutritional status was assessed with the MNA which consists of 18 items including anthropometric measurements as well as question on diet, appetite, health and disabilities (31). The score ranges from 0 to 30. A score below 17 indicates undernutrition, a score of 17–23.5 indicates a risk of undernutrition, and a score of 24 or higher indicates a satisfactory nutritional status.

BMI was calculated as body weight (kg) divided by height (m) squared. Body weight was imputed by weight measurement completed during the interview for community-dwelling participants or by a measurement of weight within 3 months for institutionalized participants. At home, body weight was measured to the nearest 0.1 kg using an electronic scale (TERRAILLON®). Participants were weighed with their clothes on, and the weight was adjusted by subtracting the average weight for the type of clothing. Height was imputed by the height value estimated from the knee-heel length using the Chumléa formula (32). Knee-heel length of the non-dominant leg was measured using a baby-height gauge with the knee and ankle joints fixed at 90° angles. A BMI lower than 21 indicates underweight, a BMI of 21–25 indicates normal weight, a BMI of 25–30 indicates overweight and a BMI of 30 or higher indicates obesity, following the recommendation of French Health Authority (29).

Background variables included age, gender, marital status, main living place during childhood, education, self-perceived financial resources, functional capacities, cognitive status and comorbidities. Regarding functional capacities, respondents completed the Short Physical Performance Battery (SPPB) which combines the results of gait speed, chair stand, and balance tests (33). The SPPB score ranges from 0 to 12 (best functional performance). Regarding cognitive status, respondents completed the Mini Mental State Examination (MMSE). The MMSE is an 11-question test that assesses five areas of cognitive function: orientation, registration, attention, recall, and language. The score ranges from 0 to 30 (best cognitive performance). Scores that are greater than or equal to 25 points (out of 30) indicate normal cognition. Below this, scores can indicate mild (21–24 points), moderate (10–20 points), or severe (≤ 9 points) cognitive impairment (34). Regarding comorbidities, respondents were asked to describe any health problems and to provide their medical prescriptions. The responses and prescriptions were then analyzed by a physician, who determined the number of comorbidities and the presence or not of a metabolic disease (*e.g*. diabetes, high blood pressure).

### Data Analysis

Descriptive data are presented as percentages or means (M) and standard errors (SE). The threshold for significance was set at 5%. For each variable, differences across weight status categories were tested using a univariate logistic regression analysis. The odds ratio (OR) and 95% confidence interval (CI) were computed for each variable associated with a significant effect. To evaluate the independence of the observed associations, the variables with a *p* value < 0.05 were simultaneously entered in a multivariate logistic regression analysis. Interaction effects were tested and removed as they were never significant. All statistical analyses were performed with SAS software (SAS Institute INC., Cary, NC, USA).

## Results

### Characteristics of the Study Sample

Seven-hundred-eighty-two participants were included in the present secondary data analysis. The characteristics of the study sample are presented in Table 1 for the whole population and for each weight status group. The respondents tended to be older, with 57% of respondents aged 80 or over. Women outnumbered men and widows outnumbered couples. Most of the respondents had a medium education level (only 20% achieved graduate school) and low to fair income (only 22% reported good income). Among the respondents, 8% were underweight, 39% overweight, and 26% obese.

**Table 1:** Characteristics of the population according to BMI status (underweight: BMI < 21; normal: 21 ≥ BMI < 25; overweight: 25 ≥ BMI < 30; obese: BMI ≥ 30).

	**Total**	**Underweight**	**Normal**	**Overweight**	**Obese**	***p*-Value^**b**^**
*N*	782	62	206	308	206	
Sex, % men	31%	32%	25%	36%	27%	0.0414
Age (yr)^a^	80.5 (0.3)	81.8 (1.1)	81.0 (0.6)	80.3 (0.5)	80.0 (0.6)	0.3618
65–80 yr	43%	42%	44%	44%	42%	0.9328
≥80 yr	57%	58%	56%	56%	52%	
Living arrangement						
At home, without help	37%	26%	39%	41%	32%	0.1004
At home, care-giver	27%	29%	25%	25%	31%	
At home, meals-on-wheels	17%	23%	21%	14%	16%	
Nursing home	19%	22%	15%	20%	21%	
Main living place during childhood					
France	97%	97%	97%	96%	98%	0.8206
Other country	3%	3%	3%	4%	2%	
Marital status						
Single	21%	39%	19%	21%	19%	0.0058
Couple	33%	21%	35%	37%	28%	
Widow	46%	40%	46%	42%	53%	
Education						
None	12%	7%	9%	12%	16%	0.0909
Primary	37%	44%	42%	32%	37%	
Secondary	31%	23%	30%	35%	31%	
Post-secondary	20%	26%	19%	21%	17%	
Income						
Low	26%	32%	23%	22%	34%	0.0596
Fair	52%	45%	54%	54%	51%	
Good	22%	23%	23%	24%	15%	
SPPB^a^	7.4 (0.1)	5.6 (0.6)	7.7 (0.3)	8.1 (0.2)	6.7 (0.3)	<0.001
MMSE^a^	27.0 (1.0)	27.4 (0.3)	27.2 (0.2)	27.1 (0.1)	26.7 (0.2)	0.1094
Comorbidities^a^	3.2 (0.1)	3.3 (0.3)	3.0 (0.1)	3.0 (0.1)	3.6 (0.2)	0.0098
Metabolic disease, %	57%	42%	48%	59%	66%	<0.001
MNA^a^						
Well-nourished (>23.5)	71%	37%	70%	80%	69%	<0.001
At risk (17–23.5)	27%	60%	27%	18%	29%	
Undernourished (<17)	2%	3%	3%	2%	2%	

*SPPB, Short Physical Activity Battery; MMSE, Mini-Mental State Examination; MNA, Mini-Nutritional Assessment*.

a *Mean ± Standard error*;

b *p-value of the Wald Chi-Square derived from a univariate logistic analysis to assess differences between weight status*.

According to univariate logistic analyses, underweight people were more likely to be single than in a couple (OR = 2.28; CI = 1.50–7.14) or widowed (OR = 2.28; CI = 1.17-4.46) compared to normal-weight individuals. In other words, the singles rate was higher in underweight people than in normal-weight people. Obese people were more likely to have a low income than a good income (OR = 2.27; Ci = 1.22–4.24) compared to normal-weight people. Obesity was also associated with a higher number of morbidities (OR = 1.15; CI = 1.04–1.27) compared to normal-weight status. Finally, people with underweight or obesity were more likely to suffer from a metabolic disease (OR = 1.56; CI = 1.09–2.23 and OR = 2.15; CI = 1.44–3.19, respectively) and they displayed a lower mobility score (SPPB) than normal-weight people (OR = 0.89; CI = 0.83–0.95 and OR = 0.94; CI = 0.90–0.99, respectively).

### Weight Status and Nutritional Status

The prevalence of undernutrition was similar across the weight status group: 3% for underweight and normal-weight respondents, 2% for overweight and obese respondents. However, being underweight was associated with the highest prevalence for the risk of undernutrition (60%). In fact, in the MNA tool, a low BMI contributes to a decrease in the global score (BMI < 19:−3 points; 19 ≥ BMI < 21:−2 points; 21 ≥ BMI < 23:−1 points) (Table 1). According to a univariate logistic analysis, underweight people were more likely to be at risk of undernutrition than well-nourished (OR = 3.61; CI = 1.98–6.58) compared to normal-weight people (*i.e*. the prevalence of people at risk of undernutrition was higher in underweight than in normal-weight people).

The risk of undernutrition ranged from 18% in overweight to 29% in obese and 27% in normal-weight participants (Table 1). According to the univariate logistic analysis, overweight people were more likely to be well-nourished than at risk of undernutrition (OR = 1.78; CI = 1.18–2.68) compared to normal-weight people (*i.e*. the prevalence of well-nourished people was higher in overweight than in normal-weight people).

To further decipher the relationship between weight status and nutritional status taking into account all the possible cofounders, a multivariate logistic analysis was performed in which weight status was a dependent variable. All the variables proved to be significantly associated with weight status in univariate analyses (*i.e*., sex, marital status, income, mobility, comorbidities, presence of a metabolic disease and nutritional status) as independent variables. Interactions were assessed and removed as none proved to be significant. When controlling for possible confounding factors, the nutritional status (MNA) remained significant (Table 2). The results confirmed that, in comparison with normal-weight respondents, being underweight was significantly associated with a higher risk of being at risk of undernutrition than well-nourished compared to normal-weight respondents. Conversely, being overweight or obese was significantly associated with a lower risk of being at risk of undernutrition than well-nourished compared to normal-weight respondents (Table 3). A closer examination of the link between BMI and metabolic disease showed that in underweight people, respondents suffering from a metabolic disease were more at risk of undernutrition than respondents without metabolic disease (OR = 4.20; CI = 1.30–13.58). However, no such difference was observed in people with overweight (OR = 0.82; CI = 0.47–1.43) or obesity (OR = 1.34; CI = 0.70–2.56).

**Table 2:** Results of the multivariate logistic regression analysis (dependent variable: weight status): Wald Chi-square and *p* value associated with each independent variable (Likelihood ratio = 166.41; *p* < 0.001).

	**Wald Chi-square**	***p*-value**
Sex	4.81	0.1862
Matrimonial status	12.16	0.0585
Income	8.60	0.1973
Mobility (SPPB)	35.15	0.6459
Comorbidities	19.56	0.9275
Presence of a metabolic disease	13.79	0.0032
Nutritional status (MNA)	32.33	<0.0001

*SPPB, Short Physical Activity Battery; MNA, Mini-Nutritional Assessment. All the variables associated with a p value < 0.05 in the univariate regression analyses were simultaneously entered in a multivariate logistic regression analysis as independent variables. Interactions were tested and removed as none proved to be significant*.

**Table 3:** Results of the multivariate logistic regression analysis (dependent variable: weight status): odds ratio estimates for the independent variable “nutritional status” (reference: normal weight).

	**Odds ratio**	**95% CI**
**Underweight**		
Undernourished versus well-nourished	1.62	0.16–16.59
At risk of undernutrition versus well-nourished	5.41	1.98–14.80
**Overweight**		
Undernourished versus well-nourished	0.15	0.02–0.96
At risk of undernutrition versus well-nourished	0.39	0.21–0.72
**Obese**		
Undernourished versus well-nourished	1.80	0.32–9.97
At risk of undernutrition versus well-nourished	0.46	0.23–0.90

## Discussion

In the older population recruited for the present experiment, 8% were underweight (8% for women and for men), 39% were overweight (36% for women, 46% for men) and 26% were obese (28% for women, 23% for men). These data are similar to those observed in the French INCA 3 study conducted in 2014–2015 among 727 people aged between 65 and 79 years old: 40% were overweight and 24% were obese (35). A study conducted in 2008 among 4 296 French older individuals aged 75 or over showed higher rate of underweight for women (20%) but not for men (8%), a similar rate of overweight (31% in women; 44% in men) and lower rates for obesity (15% in women; 14% in men) (36). Finally, according to the recent data from Eurostat (2) for France, 2% of people aged 65 or over are underweight, 38% are overweight and 17% are obese. In all of these studies, overweight was defined as a BMI between 25 and 30 kg and obesity as a BMI over 30, while the threshold for underweight varied [BMI lower than 21 in Vernay et al. (36) and in the present study; lower than 18 in Eurostat, 2021], which may explain the different rates of underweight.

Regarding nutritional status, the present study showed that 2% of the respondents with a BMI over 25 were undernourished (no difference was observed between overweight and obese people) and 23% were at risk of undernutrition (18% in overweight to 29% in obese). These features are similar to those reported by Klee Oehlschlaeger et al. (26) who observed that 23% of the seniors with a BMI over 25 were identified as at risk or undernourished by the MNA (Brazilian study), but lower than the ones reported by Özkaya and Gürbüz (27). In this Turkish study, it was observed that 19% of seniors with a BMI over 25 were undernourished and 31% were at risk of undernutrition.

According to recent census data (37), the French population has around 13,453 million people aged 65 or over. If the rates reported by Eurostat (2) for overweight and obesity in France are applied to this population, there are 5.112 million seniors are classified as overweight and 2.287 million seniors are classified as obese. Finally, when the undernutrition and risk of undernutrition rates observed in the present study are applied, there is a total 1.022 million overweight seniors and 0.709 million obese seniors are undernourished or at risk of undernutrition. In other words, it can be estimated that in France, around 1.7 million French people aged 65 or over with a BMI over 25 are undernourished or at risk of undernutrition according to the MNA. If the same iteration is applied for people with a BMI lower than 21, it can be estimated than there are around 300 000 underweight seniors who are undernourished or at risk of undernutrition in France (underweight rate was set at 8%, as observed in the present study).

According to the present results, underweight people were more likely to be at risk of undernutrition than well-nourished compared with normal weight status. In fact, a low BMI is a criterion that is used in several undernutrition screening tools including the Mini-Nutritional Assessment - MNA (31), the Nutritional Risk Screening - NRS (38) and the Undernutrition Universal Screening Tool - MUST (39), though with slightly different BMI thresholds [<19, (19–21), (21–23) for the MNA; < 20.5 for the NRS; <18.5; [18.5–20] for the MUST]. Recently, the European Society for Clinical Nutrition and Metabolism (ESPEN) agreed to consider a BMI <22 as a phenotypic criteria for the diagnosis of undernutrition in people older than 70 years (40). Conversely, overweight and obesity were associated with a lower undernutrition risk compared with normal weight status. However, older people are particularly susceptible to the adverse effects of excess body weight on physical function because of 1) a progressive loss of muscle mass and redistribution of body fat, which leads to sarcopenic obesity (41), and 2) a need to carry greater body mass due to obesity (42). In addition, obesity may result in undernutrition being overlooked in these patients (27), and the loss of muscle mass may go unnoticed until the individual begins to lose physical function (43). Stenholm et al. (44) underlined the importance of recognizing older obese persons with decreased muscle mass or strength, and suggested that clinicians and scientists needed “to identify new targets for prevention and cure of this important geriatric syndrome”. However, future researches are needed to identify which factors lead to undernutrition in obese and overweight older people compared to normal-weight or underweight people, such as the impact of metabolic disorder on protein-anabolic pathways, the impact of gut microbiota or the BMI trajectories over the life course. In fact, studies have suggested that accelerated loss of muscle mass in older adults with diabetes is mediated by a direct effect of diabetes on skeletal muscle (45, 46). It is also acknowledged that obesity is associated with alterations in the composition and diversity of the gut microbiota, which may have an impact on the absorption, storage, and expenditure of energy obtained from dietary intake. However, the extent to which this disruption in the microbial composition influences the nutritional status in obese older people has to be elucidated (47, 48). Finally, some authors have highlighted the importance of studying the life trajectory of the BMI on possible adverse outcomes (49–51). In those studies, higher frailty and mortality rates were observed in men who were overweight in midlife and in later age. However, higher frailty and mortality rates were also observed men who were overweight in midlife but were reclassified as normal-weight in later life because of weight loss, when compared to men who were constantly normal-weight. In the present experiment, it would have been of great interest to investigate nutritional status as a function of weight trajectories and not only as a function of weight status in old age.

### Study Limitations

The first limitation of the present study lies in the exclusion of older people with severe cognitive impairments. In France, it is estimated that 4% of people aged 65 or over suffer from a neurological disorder (*e.g*. Alzheimer's disease, Parkinson's disease) (52). However, it was not possible to include these people in the surveys to ensure the reliability of data collected from participants. People suffering from cognitive disorders were included in the “nursing home” category from the RENESSENS survey, but their data were excluded from the present secondary analysis to homogenize the inclusion criteria between the two surveys. However, cognitive disorders are often associated with feeding difficulties and changes in eating behavior which may cause a decrease in food intake (53–55) and altered nutritional status (56, 57). Our decision to exclude individuals with severe cognitive impairments may thus have led to an underestimation in the prevalence of undernutrition and risk of undernutrition.

A second limitation lies in the use of the Mini-Nutritional Assessment questionnaire to assess nutritional status. Recently, Cederholm et al. (40) suggested that undernutrition could be diagnosed and graded using three phenotypic criteria (non-volitional weight loss, low body mass index, and reduced muscle mass) and two etiological criteria (reduced food intake or assimilation, and inflammation or disease burden). However, at the time of the surveys, this new approach had not been yet published, and the MNA was recognized as an appropriate tool to screen for undernutrition and risk of undernutrition (58). In addition, it should be noted that Cederholm et al. (40) agreed on the use of a validated questionnaire such the MNA as a first-line screening tool to identify “at risk” status (59).

A third limitation is the cross-sectional nature of the present experimental design and the absence of data regarding the weight of the respondent at midlife. Some authors have highlighted the importance of studying the life trajectory of the BMI on possible adverse outcomes (49–51). In those studies, higher frailty and mortality rates were observed in men who were overweight in midlife and in later age. However, higher frailty and mortality rates were also observed men who were overweight in midlife but were reclassified as normal-weight in later life because of weight loss, when compared to men who were constantly normal-weight. In the present experiment, it would have been of great interest to investigate nutritional status as a function of weight trajectories and not only as a function of weight status in old age.

## Conclusion

This secondary analysis of two datasets collected from French old respondents showed that 2% of the respondents with a BMI over 25 were undernourished (no difference was observed between overweight and obese people) and 23% were at risk of undernutrition (18% in overweight to 29% in obese). Taking into account recent French census data, it can be estimated that in France, around 1.7 million people aged 65 or over with a BMI over 25 are undernourished or at risk of undernutrition. Given the growing number of overweight and obese individuals worldwide, the issue of undernutrition in the overweight/obese older population is set to become a major public health issue in the near future.

## Data Availability Statement

The datasets presented in this study can be found in online repositories. The names of the repository/repositories and accession number(s) can be found below: https://doi.org/10.15454/MJTRXM.

## Ethics Statement

The studies involving human participants were reviewed and approved by CPP Est 1 - AUPALESENS: #2010-A01079-30; RENESSENS: #2014-A00775-42. The patients/participants provided their written informed consent to participate in this study.

## Author Contributions

CS-R, VV, and IM: conceptualization, research, resource provision, and data collection. CS-R: data analysis and writing original version. VV and IM: review and correction. All authors contributed to the article and approved the submitted version.

## Funding

The surveys were funded by the French National Research Agency: [ANR-09-ALIA-011-02 and ANR-13-ALID-0006-02]. The present work also received funding from ANR (ANR-20-HDHL-0003 FORTIPHY), Research Council Norway (RCN 321819), BBSRC (BB/V018329/1) under the umbrella of the European Joint Programming Initiative A Healthy Diet for a Healthy Life (JPI HDHL) and of the ERA-NET Cofund ERA-HDHL (GA N°696295 of the EU Horizon 2020 Research and Innovation Programme).

## Conflict of Interest

The authors declare that the research was conducted in the absence of any commercial or financial relationships that could be construed as a potential conflict of interest.

## Publisher's Note

All claims expressed in this article are solely those of the authors and do not necessarily represent those of their affiliated organizations, or those of the publisher, the editors and the reviewers. Any product that may be evaluated in this article, or claim that may be made by its manufacturer, is not guaranteed or endorsed by the publisher.
